# Assessment of the Efficiency of Chemical and Thermochemical Depolymerization Methods for Lignin Valorization: Principal Component Analysis (PCA) Approach

**DOI:** 10.3390/polym14010194

**Published:** 2022-01-04

**Authors:** Khaled Younes, Ahmad Moghrabi, Sara Moghnie, Omar Mouhtady, Nimer Murshid, Laurent Grasset

**Affiliations:** 1 College of Engineering and Technology, American University of the Middle East, Kuwait; ahmad.moghrabi@aum.edu.kw (A.M.); Sarah.Mughneyah@aum.edu.kw (S.M.); omar.mouhtady@aum.edu.kw (O.M.); nimer.murshid@aum.edu.kw (N.M.); 2 Université de Poitiers, IC2MP, UMR CNRS 7285, 4 rue Michel Brunet, TSA 51106, CEDEX 9, 86073 Poitiers, France; laurent.grasset@univ-poitiers.fr

**Keywords:** lignin valorization, peatland, CuO–NaOH oxidation, thermochemolysis, principal component analysis

## Abstract

Energy demand and the use of commodity consumer products, such as chemicals, plastics, and transportation fuels, are growing nowadays. These products, which are mainly derived from fossil resources and contribute to environmental pollution and CO2 emissions, will be used up eventually. Therefore, a renewable inexhaustible energy source is required. Plant biomass resources can be used as a suitable alternative source due to their green, clean attributes and low carbon emissions. Lignin is a class of complex aromatic polymers. It is highly abundant and a major constituent in the structural cell walls of all higher vascular land plants. Lignin can be used as an alternative source for fine chemicals and raw material for biofuel production. There are many chemical processes that can be potentially utilized to increase the degradation rate of lignin into biofuels or value-added chemicals. In this study, two lignin degradation methods, CuO–NaOH oxidation and tetramethyl ammonium hydroxide (TMAH) thermochemolysis, will be addressed. Both methods showed a high capacity to produce a large molecular dataset, resulting in tedious and time-consuming data analysis. To overcome this issue, an unsupervised machine learning technique called principal component analysis (PCA) is implemented.

## 1. Introduction

The scientific community is constantly on the lookout to find alternative energy sources due to the trend in decreasing fossil energy consumption and increasing environmental pollution [1,2,3]. Researchers have been very interested in biomass resources due to their green, renewable, and clean attributes. Lignin is a component of biomass resources that contains aromatic rings [1,2,3]. Lignin has versatile applications in many areas, including chemical engineering, materials, and food. It has been a hotspot for the production of high-value chemicals and biofuels, particularly in the context of “lignin first” [4,5,6].

**Figure 1:**
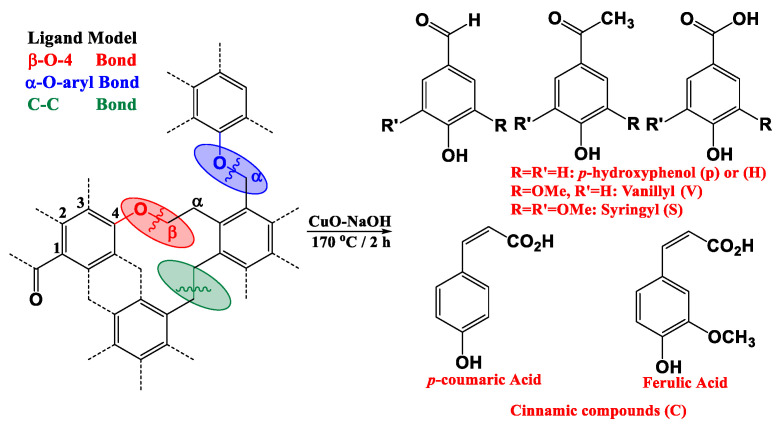
Targeted bonds and methoxyphenol compounds yielded by copper (II) oxide, alkaline oxidation [9].

**Figure 2:**
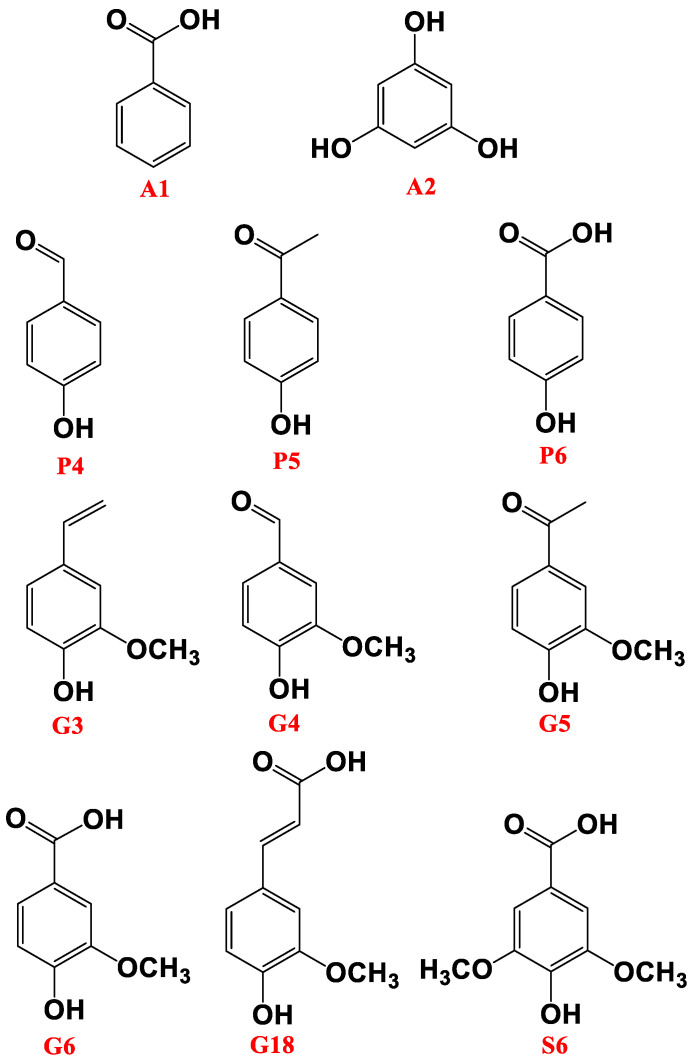
Methoxyphenol compounds yielded by TMAH themrochemolysis on the investigated peat samples [9].

Lignin is the major and most refractory counterpart of soil and plants in the geosphere. It is present in the cell walls of terrestrial higher plants [7] and can account for 15 to 35% of the dry weight of vascular land plants [8,9]. Guaiacyl (G type units), syringyl (S-type units), and p-coumaryl (H-type units) are the primary structural units of lignin. Three phenylpropane units can be linked using ether linkages and/or C–C linkages (see Figure 1) to create aromatic biomacromolecules that have three-dimensional spatial structures. The main ether linkages are β-O-4, α-O-4, γ-O-4, and 4-O-5 (Figure 1 and Figure 2). The dominant β-O-4 bonds make up more than half of the bond structure of natural lignin [10]. The C–C single bond linkage mainly consists of β-β and β-5. Because of the complex and refractory structure of lignin, its depolymerization is tedious and time-consuming. Figure 1 and Figure 2 show possible depolymerization products, produced from different degradation processes, oxidation and themrochemolysis, respectively.

There are many chemical processes that can be used to increase the conversion rate of lignin into biofuels or value-added chemicals. These include catalytic hydrogenolysis [11,12,13], catalytic oxidation [14,15,16], photocatalysis [6], pyrolysis [3,17], and electrochemical oxidation [6]. Lignin oxidation is extensively employed to create reactive lignin fragments. Upon the cleavage of the ether bonds, interesting moieties of this macromolecule are produced [18]. While oxidation has the advantage of being simple, it is also easy to modify and optimize the reaction conditions. However, product selectivity cannot be controlled [14]. Lignin oxidation can be catalyzed by metal hydroxides or salts, oxides, and complexes [6,14,18]. Many studies have shown that small molecules, such as phenolic aldehydes and ketones, are the most desirable products [6,14]. Another degradation process is pyrolysis. Lignin pyrolysis consists of the thermal degradation of the macromolecule into smaller moities. This process takes place in a free oxygen environment with and without a catalyst (mostly solid material) [6,9,17].

In this study, our aim is to shed light on two lignin degradation methods: CuO–NaOH oxidation [19,20] and the tetramethyl ammonium hydroxide (TMAH) thermochemolysis methods [9,21,22]. To our knowledge, these techniques have only been implemented for analytical uses as they showed a high capacity to extract a large molecular fingerprint [19,21,22]. Herein, we will try to seek the capacity of each technique by the application of an unsupervised machine learning technique, the so-called principal component analysis (PCA). It is expected that PCA will reveal different trends and patterns for the large molecular dataset and, therefore, can be used as a tool to probe the efficiency of the investigated method on the one hand and to decipher their range of applicability on the other hand. An overview of the two methods by a data mining technique is, interestingly, a primordial step in further optimization of the procedures and probably a scale-up of the investigated approaches.

## 2. Materials and Methods

### 2.1. Peatland Samples

The choice of peatland samples for the investigation of the efficiency of lignin depolymerization techniques has a two-folded purpose. First, lignin comprises 50 to 90% of its dry weight as organic matter (OM) [23,24,25]. In our case, the dry weight of samples yielded values of 60 to 85% in OM composition following the application of the loss of ignition (LOI) [26]. Second, the “*diplotelmic*” character of the peat splits the peat core into an upper and bottom part [26,27,28]. The upper one composes the “oxic” layer of the peat core, where microbial activity occurs and, therefore, decomposition of OM exists. This layer is the acrotelm, which is at the top of the water table (which explains the access of oxygen). The bottom part composes the “anoxic” layer of the peat core, where microbial activity does not occur and, therefore, OM decomposition is restricted to minor anaerobic reworking (e.g., sulfate-reducing bacteria). This layer is the catotelm, which is at the bottom of the water table (which explains the lack of oxygen). Other studies implemented that a transition phase between both layers can be found, the mesotelm. This layer is emerged during the summer and submerged during the winter. For this reason, the mesotelm composes a peculiar niche of microbial reworking, where facultative anaerobic strains are mostly found [26,27,28]. This division of peat core presents a source of discrepancy in the molecular composition of OM. For the investigated samples, the prevalence has been identified for all three layers [9,22,26,29]. Fresh OM (therefore, lignin) is found in the acrotelm due to the continuous input of vegetation debris from the uppermost vegetation. For the catotelm, a more degraded lignin is observed as peatland is a niche for the preservation of previously reworked OM. In the mesotelm, an intermediate state between fresh and degraded lignin has been noticed, and this is due to the intermediate position of the layer [26,27,28]. In brief, this ecological division of the investigated peatland yields the occurrence of three different stages of OM maturity. The fresh lignin is, therefore, found in the acrotelm. The degraded lignin in the catotelm and an intermediate maturity lignin is found in the mesotelm. Having this multitude of types of lignin will help to decipher which of the methods is more suited for which type of lignin.

### 2.2. Sampling Strategy

The studied peat samples are a part of the Sagnes peatbog, which has been described in previous studies [30,31,32,33]. It is in the Limousin region (central France), where igneous rocks, such as granite, dominate the subsurface (see Younes et al. [26] and Boekhout et al. [33] for a typical description). It is a river fed, topogeneous peat bog with a stream (Ruisseau des Sagnes) flowing through the topsoil. Three 1 m peat cores were collected in November 2012. Each core represented the vertices of an equilateral triangle with sides 2 m long. The cores were subdivided into 4 cm slices and freeze-dried. Five samples were collected from the upper oxic part (acrotelm; 0 to −20 cm), eight from the water table zone (mesotelm; −20 to −50 cm), and eleven from the anoxic part (catotelm; −50 to −100 cm) (Figure 2). CuO–NaOH oxidation for lignin analysis was carried out for each sample from the three cores. LOI, elemental analysis (C, H, N, and O) and TMAH thermochemolysis were carried out after combination of the samples from the three cores at the same depth.

### 2.3. Molecular Analyses

The investigated two methods are the CuO–NaOH oxidation of lignin and the TMAH thermochemolysis. Both methods have been extensively explained in previous studies performed on the same samples [22,26]. The alkaline oxidation of lignins, in the presence of copper (II) oxide, has been proposed by Hedges and Ertel [19,20]. Briefly, peat samples were mixed with CuO (II) (Sigma-Aldrich, St. Louis, MI, USA) and the mixture was put into a closed Teflon reaction (Parr instrument, Moline, IL, USA) in the presence of NaOH (1 M; Sigma-Aldrich, St. Louis, MI, USA). The reaction temperature and time are 170 °C and 2 h, respectively. The adopted procedure yielded 11 monophenolic moieties, originating from the G-, S-, and H-compounds that constitute the bricks of lignin (See introduction). The different molecules have been characterized by gas chromatography (GC; Shimadzu, Kyoto, Japan).

For TMAH thermochemolysis, peat samples were soaked with a solution of TMAH/MeOH (50/50; v/v; Sigma-Aldrich, St. Louis, MI, USA) in a furnace. The furnace was put on 400 °C for 30 min. Helium inert gas was adopted as the driving gas. The yielded pyrolysates were condensed in a cold recipient of tricholormethane. TMAH is used due to its alkalinity (pkb = 4.2) and its methylating capacity. Therefore, all labile hydrogens (alcohols and carboxylic acids) have been methylated, making the chromatographic characterization easier. The different molecules have been characterized by GC/MS (Thermo Fisher Scientific, Waltham, MA, USA).

### 2.4. Principal Component Analysis (PCA)

The principal component analysis (PCA) approach was implemented in the study analysis presented in this work. PCA was first introduced in 1901 by Karl Pearson [34,35], where it is also referred to and known as discrete Karhunen–Loève transform (KLT). PCA technique is employed to reduce the dimensionality of the problem by transforming a large set of input variables into smaller features referred to as principal components (PCs) [36]. However, reducing the number of variables might affect the accuracy, and, hence, there is a trade-off between accuracy and simplicity of the problem. Yet, the key features and the primary aim of the PCA approach is to simplify the problem by reducing the number of variables while maintaining most of the information of the input features to preserve as much accuracy of the problem as possible. PCA creates new uncorrelated variables (the PCs) in a statistical process that performs an orthogonal transformation of the possibly correlated input features. In PCA, the dimensionality of the problem is reduced by projecting the observation data orthogonally onto a lower-dimensional subspace called PCs to attain a lower-dimensional problem while preserving the accuracy as much as possible. The PCs are defined as directions that maximize the variance of the projected observation data along the principal subspace, which minimizes the loss of information and does not impact the accuracy heavily. PCA technique can be summarized in five main steps [34,35]. The primary aim of the first step is to standardize the range of the initial dataset by transforming it to comparable scales to prevent any bias in the results due to the large range since the latter statistical procedure is very sensitive and affected the variance of the initial variables (basically, variables with small ranges will be dominated by large range variables). In the second step, the covariance matrix between the different variables is computed to identify the relationship and correlation between the input variables. Then, the PCs are identified by computing the eigenvectors and eigenvalues of the covariance matrix. It can de be demonstrated that PCs are eigenvectors of the data’s covariance matrix. PCs are ranked in an order depending on the variance of the projected data. The PCA seeks to set the maximum possible information in the first PC, where it is equivalent to the direction that maximizes the variance of the projected data. Then, the next maximum remaining is set in the second PC and so on. Accordingly, in the fourth step, a feature vector set is created to decide which PC to keep and eliminate the rest in order to reduce the dimensionality of the problem. Finally, transform the original matrix where the data observation is expressed along the newly defined variables, which are the PCs axes. The last step aims to use the features vector set created using the eigenvectors of the covariance matrix to transform initial observation data from the original frame of reference to the one represented by the PCs [34,35]. In this study, XLSTAT 2014 software (Addinsoft, Paris, France) has been employed to perform PCA, and the strategy followed is the same as Younes et al. [9,22,26,29,37].

## 3. CuO–NaOH Oxidation

Alkaline oxidation in the presence of cupric oxide presents the most conventional technique for lignin molecular characterization in soils and sediments [19]. It was first implemented by Hedges and Ertel [19] and has been extensively used to decipher the molecular trends of complex organics matter matrices (soils, sediments, plant leaves).

Several distinctive indicators of lignin’s alteration state were adopted based on the relative distribution of its yielded phenolic moieties via CuO–NaOH oxidation. The application of CuO–NaOH yields to the formation of 11 monomeric phenolic moieties (Vanilic (V) compounds, Syringylic (S) compounds, and Cinnamylic (C) compounds; Figure 1). Usually, the sum of the yielded phenolic compounds decreases with the increasing degradation and/or incorporation of lignin into the so-called humic fraction. However, the authors more likely preferred some specific ratios, rather than V + S + C content, given that the yield of CuO–NaOH oxidation might vary following the degree of lignin structure alteration and oxidation [38] and following species change [39]. Along its capacity to cleave the most abundant β-O-4 aryl bonds [40], CuO–NaOH showed high efficiency in cleaving the C_α_–C_β_ bond of the phenylpropanoid [9,19,20,41,42,43,44].

## 4. Thermally Assisted Hydrolysis and Methylation (THM)

Thermochemolysis is also known as thermally assisted hydrolysis or methylation (THM). It is an established method for characterizing complex and difficult samples. In the 1960s, Downing [45] implemented this method to characterize fatty acids in the presence of tetramethylammonium hydroxyide (TMAH) by gas chromatography (GC). Challinor [46] improved the technique in 1989 and called it thermally assisted hydrolysis methylation (THM). Steinberg et al. [47] also used chemopyrolysis, yet the term thermochemolysis is more commonly used today. Thermochemolysis presents a unique in situ method for the analysis of a wide variety of materials and is becoming more popular. The heat is used to drive the reaction between the analyte’s functional group and a thermochemolysis reagent producing alkyl esters and ether groups. Heat can also be used to induce the thermal bond cleavage (in the absence of oxygen) of certain chemical bonds, such as ester bonds and ether bonds. Thermochemolysis results in the formation of products of smaller molecular weight that are less polar and alkylated and are more suited for GC analysis. Analytical pyrolysis (Py) is different from thermochemolysis in the sense that no chemical reagent is added at first. Analytical pyrolysis is the controlled thermal fragmentation and removal of complex materials in the absence of oxygen at temperatures ranging from 400 °C to 700 °C. It can be used to analyze organic compounds that are not volatile or insoluble. It is, therefore, suitable for the direct analysis of solids using common analytical techniques, such as GC and GC/MS. One of the major shortcomings of this method is the inability to control reactions for complex molecular structures. In other words, side reactions are more likely to occur, and the most pronounced of them is the dehydration of hydroxyl groups to yield alkenes and the decarboxylation of yielded fatty acids from complex lipid structures [21]

Both thermochemolysis and analytical pyrolysis deal with the chemical alteration of materials that have complex or polymeric structures. The chemical analysis is based on the observed products and aims to chemically profile, structurally characterize, and determine the origin of the input material. Analytical pyrolysis can result in more complex fragmentation patterns and higher amounts of pyrolysates due to the increased energy input and different rates of decomposition [9,21,22]. The advantage of thermochemolysis is that it can be more selective in the cleavage and fusion of ester and ether bonds. This process can be performed at lower temperatures with less thermal fragmentation. In addition, no product derivation is taking place in analytical pyrolysis yielding for compounds that have poor GC behavior [9,21,22].

Several thermochemolysis studies are primarily concerned with the analysis and investigation of chemical bonding among lignin moieties. Kuroda et al. [48,49,50,51] applied thermochemolysis for the sake of comprehensively investigating the lignin structural network based on the unique chemical bonds between subunits. A study of the β-5 behavior of the lignin model compound showed that synthetic lignins have high levels of terminal substructures [48], while naturally occurring lignin, such as cedar softwood, has very few of these substructures [48]. A study on thermochemolysis of softwood lignin suggested that 1-(3,4-dimethoxyphenyl)-2-methoxyethene and 1-(3,4-dimethoxyphenyl)-1,2,3-trimethoxypropane are produced from β-aryl ether subunits [50]. This work used a combination derivatization technique as methylation was combined with acetylation. This coupling was intended to have extensive structural elucidation of the lignin molecular composition. Lignin model compounds containing β-β linkages, such as pinoresinols and syringaresinol, were subjected to thermochemolysis-TMAH, and unique products of di-O-methylpinoresinol and di-O-methylsyringaresinol were formed, respectively [52]. The main mechanistic step in the thermochemolysis was the opening of the aryl ether linkage. The thermochemolysis products also included diphenylpropane derivatives and stilbene-type products.

The yielded products of the thermochemolysis in the presence of TMAH, shown in Figure 2, were compared with the CuO–NaOH oxidation method [53,54] for the depth records of an ombotrophic peatland. It has been found that CuO–NaOH was more suited to low degraded less-oxidized lignin moieties due to its capacity to cleave both C–C and C–O bonds across the polymeric structure [9]. On the other hand, thermochemolysis, with the adopted conditions, showed a high capacity to exclusively cleave C–O lignin, making it more suited for more oxidized lignin moieities [9]. Following this principle, thermochemolysis was recommended to be more likely applied for fossilized sediments rather than CuO–NaOH oxidation, which should be dedicated to recent sediments or for the analysis of plant leaves. One of the shortcomings of both methods is their capacity to yield a large molecular fingerprint. Hence, analyzing and inspecting molecular degradation methods is found to be tedious and time-consuming. One way to overcome this issue is to adopt a data-reduction approach. Principal component analysis (PCA) is one of the most efficient and, therefore, one of the most applied techniques. In this study, we will apply PCA to the molecular fingerprint of both methods and attempt to reveal which of these methods is more suited, following the degree of maturation of the three types of lignins, distributed along the ecological layers of the investigated peat core.

## 5. Results and Discussion

### 5.1. PCA CuO–NaOH

**Figure 3:**
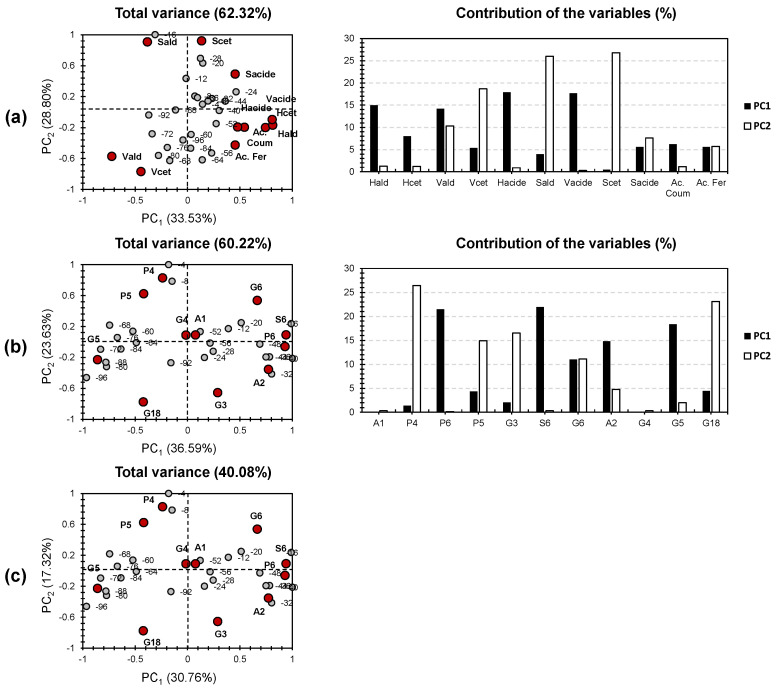
PCA for the molecular dataset yielded by the investigated techniques: (**a**) CuO–NaOH oxidation, (**b**) TMAH thermochemolysis, and (**c**) Total dataset.

The first two components, PC1 and PC2, accounted for 62.32% of the total variance (Figure 3). The 24 analyzed samples were gathered into two clusters that present the diplotelmic (two layers) character of a peatland. PC1 and PC2 separately explained 33.53% and 28.80%, respectively. The samples from −4 to −52 cm, clustering in the top region, are positively correlated with PC1. The samples of the bottom half (Catotelm; from −50 cm to the bottom) are located in the bottom part of the PCA plot. It is worth mentioning that all the depth records were dispatched in a minor way relative to PC2.

For the variables, Vald presented high influence on both PCs (around 10–15%). Hald, Hacid, and Vacid presented high influence on PC1 (around 15–20%). As for PC2, Vcet, Sald, and Scet were the most dominant contributors (around 15–30%). Three different variable clusters can be distinguished in respect to the first two PCs. The first cluster, regrouping S-compounds, presented high positive influence for PC1 and was located along with the mesotelm depth records (−12, −6, −20, and −28 cm). The second group, regrouping Vald and Vket, presented high negative influence on both PCs and was located along the deepest depth records of the peat core. Interestingly, Vacid was excluded from the second cluster as it formed along with H- and C-compounds, the third cluster. The latter presented a positive influence along PC1 and a negative one along PC2 and was located along the upper depth records of the catotelm (interface between the mesotelm and catotelm).

The double clustering effect, noticed for CuO–NaOH, indicates the capacity of this method to only decipher the upper and bottom depth records, excluding the intermediate transition phase between the “oxic” and “anoxic” parts of the peatland. Interestingly, the mesotelm depth records were clustered along with the acrotelm layer. This indicates that the “fresh” lignin (from acrotelm) and the slightly reworked lignin (from mesotelm) are targeted by CuO–NaOH, with the same efficiency. For these two types of lignin, the production of S-, H-, and C-compounds is more efficient than G-compounds. The latter requires a more reworked lignin (presented by catotelm samples). Hence, G-compounds could be more likely yielded by the application of CuO–NaOH in waste treatment and valorization as more reworked macromolecules will be in hand.

### 5.2. PCA TMAH

The first two components, PC1 and PC2, accounted for 60.22% of the total variance (Figure 3). This value is, interestingly, similar for the case of CuO–NaOH, indicating the equal reliability of PCA in treating datasets of the investigated lignin depolymerization methods. The 24 analyzed samples were gathered around into three clusters that compose the ecological layer composing a peatland. PC1 and PC2 separately explained 36.59% and 23.63%, respectively. The uppermost depth records (−4 and −8 cm) were separately clustered from other depth samples and contributed a high positive impact by PC1, and a slightly negative impact by PC2. The second cluster presented a positive influence with respect to PC1. For PC2, the cluster was sub-divided into two groups, between slight positive influence for one group and slight negative influence for the other. Interestingly, the positive PC2 influence was most likely noticed for the bottom acrotelm depth samples (−12, −16, and −20, representing the interface between acrotelm and mesotelm), and the negative influence was noticed for the mesotelm depth samples. This separation of the second cluster was noticed for the third one, regrouping the catotelm. For this layer, all the samples were negatively influenced by PC1. The depth records of the upper part of the catotelm (−60 and −68 cm) were positively influenced by PC2; as for the bottom part, more likely, a negative influence has been observed.

For the variables, G6 presented high influence on both PCs (around 10%). P6, S6, A2, and G5 presented high influence on PC1 (around 15–25%). As for PC2, P4, P5, G3, and G18 were the most dominant contributors (around 10–30%). A1 and G4 presented centered positions along PC1 and PC2, indicating their minor influence on the dataset. Three different variable clusters can be distinguished in respect to the first two PCs. These trends are in accordance with the presence of three different clusters for the individuals (depth peat samples).

The first cluster, regrouping P4 and P5, presented high positive influence for PC1 and was located along with the acrotelm depth records (−4 and −8 cm). The second group, regrouping A1, P6, G3, S6, G4, A2, and G6, presented high positive influence on PC1 and either slight positive or negative influence in respect to PC2. The third cluster, formed by G5 and G18, presented negative influence on both PCs and was located along with the mesotelm records.

The triple clustering effect, noticed for TMAH, indicates the capacity of this method to decipher all three ecological layers. This indicates that the “fresh” lignin (from acrotelm) and the slightly reworked lignin (from mesotelm) are targeted by TMAH with different efficiency. Unlike CuO–NaOH, the TMAH method showed high distribution of the phenolic sub-units. In other words, no clustering of the A-, P-, G-, and S-compounds was noticed. This indicates that, even though TMAH thermochemolysis showed a higher efficiency in targeting more types of lignins (three clusters rather than two in the case of CuO–NaOH), its operational conditions need to be enhanced. This high bias in the case of TMAH could be explained by the pyrolytic conditions. In fact, high temperatures increase the possibility of side-reactions and unexplained interactions. On the other hand, TMAH is a highly reactive ionic liquid and is, therefore, highly unstable. It shows a high capacity of degradation into trimethylamine and methanol. This may shift the reaction to the purely pyrolytic path.

### 5.3. PCA for the Total Molecular Dataset (TMAH and CuO–NaOH)

The first two components, PC1 and PC2, accounted for 48.08% of the total variance (Figure 3). This value is slightly smaller than the case of CuO–NaOH and TMAH when treated individually. This could be due to the mechanistic difference in each of the investigated methods. Interestingly, similar clustering to the one revealed, in the case of TMAH, is present. This could be explained by the higher contribution of TMAH factors, if compared with the contribution of the yielded moieties, in the case of CuO–NaOH. In fact, the 24 analyzed samples were gathered into three clusters that compose the ecological layer of the studied peatland. PC1 and PC2 separately explained 30.76% and 17.32%, respectively. The uppermost depth records (−4 and −8 cm) were separately clustered from other depth samples and contributed a high positive impact by PC1, and a slightly positive impact by PC2. The second cluster presented a positive influence with respect to PC1. For PC2, the cluster was sub-divided into two groups, between slight positive influence for one group and a high negative influence for the other. Interestingly, the positive PC2 influence was most likely noticed for the bottom acrotelm depth samples (−12, −16, and −20, representing the interface between acrotelm and mesotelm), and the negative influence was noticed for the mesotelm depth samples. This separation of the second cluster was noticed for the third one, regrouping the catotelm. For this layer, the interface mesotelm-catotelm samples (−52 and −56 cm) presented nearly negligible influence by both PCs. The other catotelm samples were negatively influenced by PC1. The depth records of the upper part of the catotelm (−60, −68, and −76 cm) were positively influenced by PC2; as for the bottom part, a negative influence has been observed.

### 5.4. Elemental Proxies and Factors Comparisons

Bulk elemental analysis methods have been employed, in our previous studies, for the sake of characterizing OM along the investigated peat core and to reveal geological and ecological answers [22,26]. The C/N ratio decreasing profile reflects the relatively higher degradation of N-poor biodegradable biopolymers (i.e., carbohydrates; [19,22,55]). For peat bulk analysis, C/N ratio has been employed as an indicator of carbon mineralization during the periods of emerged upper half (the lowering of water level in the mesotelm) [22,25]. In our case, the C/N ratio could be employed for estimating the prevalence between the refractory OM compartment (lignin and lipids, and represented by an increase in the amount of C) in one part, and the labile OM compartment (carbohydrates and amino acids, and represented by an increase in the amount of N) in another part. Following the previous statements and considering the low C/N values scored in the upper part of the peat column (<25) and the increasing that occurred in the low part (around 50), C/N can be regarded as a lignin dynamics indicator. H/C ratio is considered as an aromaticity index [26,56]. It ranged, in our case, from 1.1 to 1.7, therefore showing minor fluctuations. O/C has been extensively employed as an indicator of the oxidation/degradation of OM [22,25,57]. In our case, O/C showed a decreasing profile along the depth of the peat core [22]. This could be explained by the variation in microbial activity from the higher “oxic” part of the peat column (acrotelm and mesotelm) to the lower “anoxic” part (catotelm).

**Figure 4:**
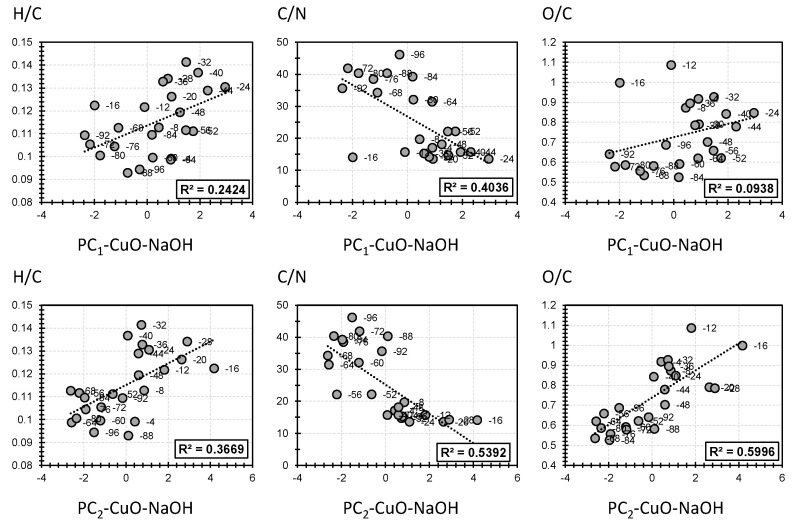
Selected correlation between bulk analysis proxies and the first two principal components (PC1 and PC2) obtained by PCA of CuO–NaOH method.

Figure 4 describes the correlation between the elemental analysis proxies H/C, C/N, and O/C, on the one hand, and the first two PCs of the molecular fingerprint yielded from the CuO–NaOH oxidation method on the other hand. PC1 showed high correlation in respect to H/C and C/N (24% and 40%, respectively; Figure 4). It is worth mentioning that H/C was directly proportional, and C/N was inversely proportional, in respect to PC1. This indicates that higher H/C and lower C/N biomass samples are more suited to be employed for the production of moieties that showed a high positive contribution to PC1. On the contrary, lower H/C and higher C/N biomass samples are more suited to be employed for the production of the moieties that showed high negative contributions in respect to PC1. Such conclusions cannot be revealed for O/C as a low correlation with PC1 has been noticed (9%; Figure 4). For PC2, it showed high correlations to all three elemental proxies. Interestingly, the highest correlation was yielded between PC2 and O/C (60%, 53%, and 37% for O/C, C/N, and H/C, respectively; Figure 4). It is worth mentioning that PC2 showed the same trends as PC1 in regard to its correlation with H/C and C/N. Hence, H/C and O/C were directly proportional and C/N was inversely proportional in respect to PC2. This indicates that higher H/C and O/C and lower C/N biomass samples are more suited to be employed for the production of the moieties that showed a high positive contribution to PC2. On the contrary, lower H/C and C/N and higher O/C biomass samples are more suited to be employed for the production of the moieties that showed a high negative contribution in respect to PC2.

**Figure 5:**
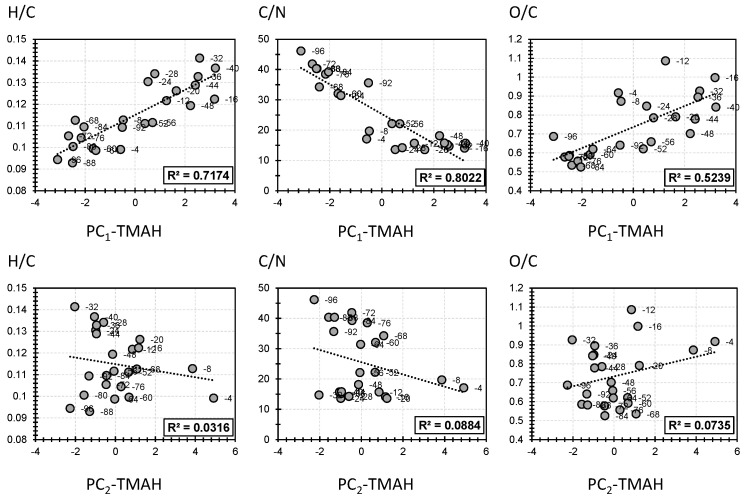
Selected correlation between bulk analysis proxies and the first two principal components (PC1 and PC2) obtained by PCA of TMAH thermochemolysis method.

Figure 5 describes the correlation between the elemental analysis proxies H/C, C/N, and O/C, on the one hand, and the first two PCs of the molecular fingerprint yielded from TMAH thermochemolysis on the other hand. PC1-TMAH showed a high correlation in respect to all three elemental proxies (71%, 80%, and 52% for H/C, C/N, and O/C, respectively; Figure 5). It is worth mentioning that a higher correlation for PC1-TMAH was yielded, if compared with the correlation of PC1-CuO–NaOH, in respect to elemental proxies (Figure 4 and Figure 5). Interestingly, similar trends of proportionality for PC1-TMAH were yielded as PC1-TMAH exhibited positive correlations in respect to H/C and C/N and negative correlations in respect to O/C (Figure 5). This indicates that higher H/C and O/C and lower C/N biomass samples are more suited to be employed for the production of the moieties that showed a high positive contribution to PC1-TMAH. On the contrary, lower H/C and O/C and higher C/N biomass are more suited to be employed for the production of the moieties that showed a high negative contribution in respect to PC1-TMAH. Such conclusions cannot be identified for PC2-TMAH as low correlations were noticed in respect to the three elemental proxies (3%, 9%, and 7% for H/C, C/N, and O/C, respectively; Figure 5).

In this part, we have attempted to decipher the scope of applicability of the two proposed techniques following the bulk analysis profile, which reflects the nature of the investigated biomass (amount of lignin (C/N), degree of oxidation (O/C), and H/C). Following the different yielded trends, it is presumed that CuO–NaOH oxidation is more suited for the sake of producing H-compounds, Vacid, Sacid, and C-compounds in the case of a biomass with low lignin content (low C/N) possessing an H/C proxy close to 1.1 and regardless of the biomass’s oxidation state. In the case of low oxidized samples, Sald and Scet will be more favored for a low lignin amount biomass (low C/N) and an H/C proxy near 1.7. For high lignin amount and low oxidized biomass (high C/N and low O/C) with an H/C proxy near 1.1, Vald and Vcet are the most favored moieties. For the TMAH thermochemolysis procedure, P4, P5, and G6 are more likely to be produced from low lignin content and highly oxidized biomass (low C/N and high O/C) with an H/C proxy near 1.7. Conversely, a high lignin content and less oxidized biomass (high C/N and low O/C) can be more implemented in cases where G3 and G18 are the targeted moieties to be produced.

## 6. Conclusions

The capacity of the lignin degradation using both CuO–NaOH oxidation and the TMAH thermochemolysis methods have been evaluated using principal component analysis (PCA). The study has been conducted on 24 peatland samples. The first two components, PC1 and PC2, showed comparable variances from both CuO–NaOH and the TMAH methods. The two PCs accounted for 62.32% and 60.22% of the total variance for CuO–NaOH and TMAH, respectively. This indicates the equal reliability of PCA in treating the datasets of the investigated lignin degradation methods. However, PCA for the total molecular dataset for both CuO–NaOH and TMAH revealed 48.08% of the total variance, smaller than the case when each method is treated individually. Even though both molecular methods showed similar variance along PC1 and PC2, high discrepancies between the peat sample distribution and factor influence were noticed. Following PCA, CuO–NaOH oxidation showed, in the case of low lignin, a better applicability if H-compounds, Vacid, Sacid and C-compounds, production is of interest. For a high lignin amount with a low oxidation state, Vald and Vcet are more likely to be produced. In the case of TMAH thermochemolysis, and for low lignin content and highly oxidized biomass, P4, P5, and G6 are more likely to be produced. Conversely, a high lignin content and less oxidized biomass can be more implemented in the case where G3 and G18 are the targeted moieties to be produced.

## Data Availability

Available upon request.
